# An investigation on the relevance of prolactin, insulin‐like growth factor‐1 and 25‐hydroxyvitamin D_3_ (25‐OHD_3_) in canine benign prostatic hyperplasia in a predisposed breed model

**DOI:** 10.1002/vms3.514

**Published:** 2021-05-20

**Authors:** Franziska Werhahn Beining, Marion Schmicke, Mirja Wilkens, Karola Wolf, Karl Rohn, Anne‐Rose Günzel‐Apel

**Affiliations:** ^1^ Unit of Reproductive Medicine – Small Animal Clinic University of Veterinary Medicine Hannover Hannover Germany; ^2^ Clinic for Cattle University of Veterinary Medicine Hannover Hannover Germany; ^3^ Institute for Physiology and Cell Biology University of Veterinary Medicine Hannover Hannover Germany; ^4^ Institute for Biometry, Epidemiology and Information University of Veterinary Medicine Hannover Hannover Germany

**Keywords:** 25‐OHD_3_, breed predisposition, IGF‐1, prevention of BPH, prolactin

## Abstract

Serum concentrations of prolactin (PRL), insulin‐like growth factor‐1 (IGF‐1) and 25 hydroxyvitamin D_3_ (25‐OHD_3_) were analysed to investigate their possible involvement in the pathogenesis of benign prostatic hyperplasia (BPH). For this, dogs of the Rhodesian Ridgeback (RR) breed were used because of a verified breed disposition for the development of BPH. Labrador Retrievers (LR) served as controls. The prostate gland status was characterised by the prostate gland volume, clinical signs of BPH (haemospermia and sonographic findings) and the plasma concentration of canine prostate‐specific arginine esterase (CPSE). Breed specificity in the RR was expressed by a correlation of PRL with breed (*p* < 0.05). Similar relationships existed in the dogs with normal CPSE (CPSEn) with respect to the IGF‐1 concentrations (LR: *p* < 0.05). The latter were negatively correlated with prostatic volume and age (both *p* < 0.05). Concentrations of 25‐OHD_3_ were tendentially (*p* = 0.18) lower in the RR with increased CPSE (CPSEi) compared with the CPSEn LR and RR showing clinical signs of BPH. A negative correlation between serum 25‐OHD_3_ and age (*p* < 0.05) existed in the CPSEi RR. Proof of 25‐OHD_3_ in prostatic secretion proved to be a breed specific feature in the RR (*p* < 0.0001). For all RR dogs showing clinical signs of BPH, a close to significant (*p* = 0.06) positive correlation with prostate gland volume was found. The results of the present study reveal no clear hints towards the significance of PRL and IGF‐1 in the pathogenesis of canine BPH. In the RR breed there were indications of a causal relationship with age‐dependent changes in the vitamin D metabolism. The data suggest the possibility of preventing or treating canine BPH by administering vitamin D or substances involved in the intraprostatic vitamin D metabolism.

## Graphical abstract

Serum concentrations of prolactin (PRL), insulin‐like growth factor‐1 (IGF‐1), and 25 hydroxyvitamin D_3_ (25‐OHD_3_) were analysed to investigate their possible involvement in the pathogenesis of benign prostatic hyperplasia (BPH) in dogs of two breeds, the Rhodesian Ridgeback (RR) and Labrador Retriever (LR). The results of the present study reveal no clear hints towards the significance of PRL and IGF‐1 in the pathogenesis of canine BPH. In the RR breed there were indications of a causal relationship with age‐dependent changes in the vitamin D metabolism. The data suggest the possibility of preventing or treating canine BPH by administering vitamin D or of substances involved in the intraprostatic vitamin D metabolism.

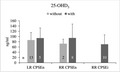

## 1 INTRODUCTION

Benign prostatic hyperplasia (BPH) is an age‐dependent enlargement of the gland and the most common prostatic disease in intact dogs (Berry, Coffey, et al., 1986; Berry, Strandberg, et al., 1986). Tissue alterations include both hyperplasia and hypertrophy of the secretory glandular epithelium and stromal components (Zirkin & Strandberg, 1984). The probability of developing BPH increases by the age of 2–3 years (Teske et al., 2002). A prevalence of 95%–100% is reached at the age of 8–9 years (Berry, Coffey, et al., 1986; Berry, Strandberg, et al., 1986; Gobello et al., 2002; Lowseth et al., 1990). An increased intraprostatic reduction in testosterone into 5α‐dihydrotestosterone is regarded as the main trigger of BPH (Bamberg‐Thalen & Linde‐Forsberg, 1993; Barsanti & Finco, 1986; Ewing et al., 1984; Gloyna et al., 1970; Isaacs & Coffey, 1981; Johnson, 2010; Walsh & Wilson, 1976).

Besides androgen dependency, other factors which may cause BPH in dogs are largely unknown. In humans, prolactin (PRL) and vitamin D have been shown to affect the prostate gland and semen quality (Adorini et al., 2010; Bachelot & Binart, 2007; Blomberg Jensen et al., 2011; De Rosa et al., 2004). In men and rats, an androgen‐dependent intraprostatic increase in PRL and its receptors is described (Hair et al., 2002; Ouhtit et al., 1993). Moreover, PRL may raise the uptake of testosterone into the prostate and the intraprostatic testosterone metabolism (Farnsworth et al., 1981).

Low vitamin D status, verified by plasma concentrations of 25‐hydroxyvitamin D_3_ (25‐OHD_3_) is considered a risk factor in human BPH (Espinosa et al., 2013). Moreover, anti‐tumour activity of the biologically active form of vitamin D, 1,25‐dihydroxyvitamin D_3_ (1,25‐(OH)_2_D_3_) acting via the vitamin D receptor (VDR), has been verified in human and rat prostatic tissue (Blomberg Jensen et al., 2011; Johnson et al., 1996; Kivineva et al., 1998).

The polypeptide insulin‐like growth factor‐1 (IGF‐1) is a strong mitogen for normal and abnormal cells. By this, it increases proliferation and decreases apoptosis and intensification of cellular differentiation (Le Roith, 1996). The impact of IGF‐1 on proliferation of many tissues was considered regarding BPH in men (Khosravi et al., 2001). Thus, an anti‐apoptotic effect of IGF‐1 on epithelial and stromal cells of the prostate acting via its membrane receptor (IGF‐R1) and tyrosine kinase was shown, leading to an increase in hypertrophic changes (Monti et al., 2001).

According to the above mentioned research data we hypothesized that canine BPH might be related to increased serum concentrations of PRL and IGF‐1 and decreased concentrations of 25‐OHD_3_. Furthermore, we expected insight into the prostatic vitamin D metabolism by measuring the 25‐OHD_3_ concentration in prostatic secretion.

The objectives of the present study was to extend the knowledge about the involvement of non‐steroidal factors like PRL, IGF‐1, and vitamin D in the pathogenesis of canine BPH. For this, the serum concentrations of the three substances and the prostatic secretion 25‐OHD_3_ concentration in two selected breeds were compared. The Rhodesian Ridgeback was chosen due to a verified breed disposition to develop BPH, and the Labrador Retriever showing species‐specific development of BPH served as control ( ).

## 2 MATERIALS AND METHODS

### 2.1 Animals and experimental design

A total of 38 physically healthy privately owned male breeding dogs of two selected breeds (Labrador Retriever/LR, *n* = 18 and Rhodesian Ridgeback/RR, *n* = 20) were included in this study. The dogs' age was 18–24 months (LR: *n* = 6, RR: *n* = 6), 25–48 months (LR: *n* = 7, RR: *n* = 6) and 49–72 months (LR: *n* = 5, RR: *n* = 8). Regarding the health status, thyroid function was additionally considered in all dogs. This was checked by measuring the serum concentrations of thyroxine and thyrotropin (TSH). Only dogs with concentrations of both hormones within the laboratory‐specific reference range were used. The RR was chosen due to a verified breed‐related increased tendency to develop BPH. The LR served as control. In the latter breed, benign enlargement of the prostate gland has been verified to occur in accordance with the results of earlier studies demonstrating expected age‐related changes in the canine prostate gland ( ). A further criterion for using dogs of these two breeds was their similar body weight (LR: 37.2 ± 4.0 kg; RR: 41.7 ± 3.7 kg) in order to allow the group‐wise comparison of obtained results. With regard to the prostate gland status, the dogs were assigned to the groups “without or with clinical signs of BPH” and with “normal serum concentration of the canine prostate‐specific arginine esterase (CPSEn)” or with “increased serum concentration of CPSE (CPSEi).”

### 2.2 Determination of the prostate gland status

The prostate gland status was characterized by morphological criteria obtained from digital rectal palpation regarding the approximate size, symmetry or asymmetry, the consistency and the position (pelvic and abdominal) as well as the presence of painfulness. In addition, sonographic images of the glandular parenchyma were made. The findings “hypoechoic, homogeneous” were assessed as “normal,” “hypoechoic, inhomogeneous” and “inhomogeneous with small cysts” as “abnormal” ( ). The sonographic presentation of the prostate gland was performed as described by Ruel et al., (1998) using the ultrasound machine Logic 5 Pro (General Electric Medical Systems GmbH, Solingen, Germany) and a 6.0 MHz micro‐convex transducer. For determining the prostatic volume, prostatic length, width and height were measured three times on maximum longitudinal and transverse sections of ultrasound images and mean values were calculated. Prostatic volume was estimated using the formula for the volume of an ellipsoid body (volume = length × width × height × 0.523) according to Ruel et al., (1998). A complete breeding soundness examination was performed to exclude dogs with testicular dysfunction and sub‐ or infertility. Semen analysis included macroscopic and microscopic criteria as described in our previous paper ( ). Only dogs with ejaculates showing normospermia or a minor dysspermia consisting of a slightly increased percentage of morphologically abnormal spermatozoa as the only alteration in semen quality were selected. To largely rule out chronic prostatitis, special attention was additionally directed towards the presence of inflammatory cells like leukocytes (Johnston et al., 2000). Moreover, one sample either of pre‐secretion (*n* = 30) or, if pre‐secretion could not be obtained, of sperm‐rich fraction (*n* = 8) from each ejaculate was subjected to microbiological examination at the Institute of Microbiology, University of Veterinary Medicine Hannover, Germany. Only dogs with no or a low to medium degree of non‐specific bacteria were included in the study.

Haemospermia, which is known to be a typical sign of canine BPH (England & Allen, 1992; Johnston et al., 2000), was verified by the macroscopic appearance (reddish colour) and the microscopic verification of erythrocytes in the third ejaculate fraction representing prostatic secretion (England & Allen, 1992). The latter was separated from the sperm‐rich fraction during routine semen collection (Günzel‐Apel, 2016). From each ejaculate, 6 ml of the prostatic secretion were transferred into a plastic tube and stored at −20°C until laboratory analysis.

In addition to the clinical signs of BPH (sonographic findings/SF and/or the presence of haemospermia/HS) the blood plasma concentration of the canine prostate‐specific arginine esterase (CPSE) was determined for classifying the prostate gland status. A CPSE concentration of ≤60 ng/ml was equated with “normal” (CPSEn), a concentration of ≥61 ng/ml with “increased” (CPSEi) based on the findings of Pinheiro et al., (2017), who validated the CPSE assay used in our study.

Blood samples were collected from the left or right cephalic vein and left at room temperature for 20 min. Blood serum was separated by centrifugation at 3,030 × *g* for 10 min. The supernatant was divided into split samples of 0.5–1.0 ml according to the number of evaluated parameters. All samples were stored at −20°C until analyses.

### 2.3 Laboratory analyses

The plasma concentration of canine prostate‐specific arginine esterase was determined using the commercial enzyme‐linked immunosorbent assay (ELISA) (Odelis®; CPSE, Virbac Tierarzneimittel GmbH). Analyses were performed in accordance with the manufacturer's instructions.

Measurement of prolactin concentrations in peripheral blood serum was realized using the Demeditec Prolactin canine ELISA (Demeditec Diagnostics GmbH) in accordance with the manufacturer's instructions. The intra‐assay coefficient of variation was 6.3%.

The IGF‐1 concentration was determined by means of the radioimmunoassay kit (IRMA) (Immunotech Beckman Coulter, CA). Analyses were carried out corresponding with the manufacturer's information. The intra‐assay coefficient of variation was 9.8%.

The concentration of calcidiol/25‐hydroxy vitamin D_3_ (25‐OHD_3_) in blood serum and prostatic secretion was determined using a commercially available competitive ELISA kit (Immundiagnostik AG) in accordance with the manufacturer's instructions.

### 2.4 Statistical evaluation

For statistical evaluation, the SAS^®^ program (Statistical Analysis System^®^, SAS Institute Inc., Version Enterprise Guide^®^ 7.1) was employed.

The dogs of either breed were grouped according to the CPSE status (normal versus. increased) and the absence or presence of clinical signs (without vs. with).

The descriptive statistic was performed by non‐parametric one‐factorial ANOVA. Wilcoxon's signed‐rank test and the Kruskal‐Wallis test were used to compare the differences in the various parameters (prostate gland volume, PRL, IGF‐1, 25‐OHD_3_) between groups regarding breed, CPSE status, and absence or presence of clinical signs of BPH. Results are presented as mean ± *SD*. Differences between groups were assessed as being significant if *p* < 0.05. The Spearman's rank correlation coefficient was determined for characterization of related influences. As one blood serum sample of an LR dog had been lost, the statistical analyses referring to PRL in the LR dogs were based on 17 instead of 18 samples.

## 3 RESULTS

### 3.1 Prostate gland status

**FIGURE 1:**
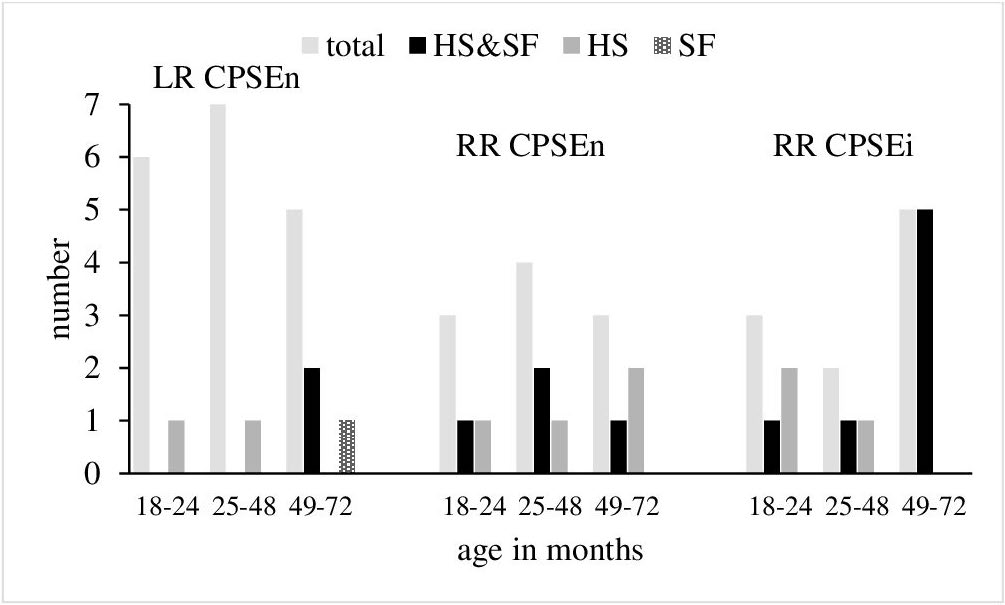
Age related distribution of dogs with normal (*n*) and increased (*i*) concentrations of canine prostate‐specific arginine esterase (CPSE) and with clinical signs of BPH (HS, haemospermia; SF, sonographic findings)

In the 38 dogs selected for the present study, the prostate gland was located in pelvic positon. In all dogs of the LR breed, the CPSE concentrations were within the normal range. Haemospermia alone was present in one dog each from age groups 18–24 months and 25–48 months. In age group 49–72 months, three dogs showed SF alone (*n* = 1) or combined with HS (*n* = 2) (Figure 1). Out of the 20 RR dogs, 50% each had normal or increased CPSE concentrations. Clinical signs of BPH were recorded in 80% of the RR with CPSEn and in 100% of the RR with CPSEi (HS alone *n* = 7, HS plus SF *n* = 11). The age‐related distribution of dogs with CPSEn and CPSEi is shown in Figure 1.

Prostate gland volume (PGV) was 26.1 ± 11.4 ccm in the LR dogs and 67.1 ± 38.2 ccm in the RR dogs irrespective of the CPSE status (*p* < 0.05). In the LR dogs, all showing normal CPSE concentrations, no difference in PGV existed between those without and with clinical signs of BPH (24.6 ± 10.0 ccm v. 30.1 ± 15.0 ccm). An almost identical PGV was found in the two CPSEn RR without clinical signs (24.4 ± 6.0 ccm). In the CPSEn RR and CPSEi RR with clinical signs of BPH, the PGV compared to the LR CPSEn with clinical signs was close to significantly larger (60.0 ± 35.1 ccm, *p* = 0.06) and significantly larger (81.3 ± 38.1 ccm, *p* < 0.05), respectively.

Regarding the entire group of dogs, PGV was correlated with the breed (*r* = 0.67, *p* < 0.0001). A correlation with age was observed for the LR without clinical signs (*r* = 0.85, *p* < 0.001), the RR CPSEn with clinical signs (*r* = 0.79, *p* < 0.05) and for all RR with clinical signs (*r* = 0.51, *p* < 0.05) (Table 1).

**TABLE 1:** Correlations of prostate gland volume (PGV), concentrations of prolactin (PRL), IGF‐1 and 25‐OHD_3_ in serum (S) or prostatic secretion (PS) with breed, age, and among themselves regarding the CPSE status and clinical signs of BPH (without, with)

	Total (CPSEn&CPSEi) (*n* = 38)		LR (CPSEn without) (*n* = 13)		RR (CPSEn with) (*n* = 8)		RR (CPSEi with) (*n* = 10)		RR (CPSEn&CPSEi with) (*n* = 18)	
	*r*	*p*	*r*	*p*	*r*	*p*	*r*	*p*	*r*	*p*
PGV	Breed									
	0.67	<0.0001	—	—	—	—	—	—	—	—
	Age									
	—	—	0.85	<0.001	0.79	<0.05	—	—	0.51	<0.05
PRL	Breed									
	0.39	<0.05	—	—	—	—	—	—	—	—
	CPSE									
	—	—	—	—	—	—	0.72	<0.05	—	—
IGF‐1	PGV									
	−0.33	<0.05	—	—	−0.89	<0.01	—	—	−0.47	=0.05
	age									
	−0.37	<0.05	—	—	−0.79	<0.05	—	—	—	—
	CPSE									
	−0.36	<0.05	—	—	—	—	—	—	—	—
S−25‐OHD_3_ PS‐25‐OHD_3_	age									
	−0.33	<0.05	—	—	—	—	−0.64	<0.05	−0.47	=0.05
	breed									
	0.59	<0.0001	—	—	—	—	—	—	—	—
	PGV									
	—	—	—	—	—	—	—	—	0.46	=0.06

Abbreviations: CPSEn ‐ normal concentration, CPSEi—increased concentration; LR, Labrador Retriever; RR, Rhodesian Ridgeback.

### 3.2 Prolactin

**FIGURE 2:**
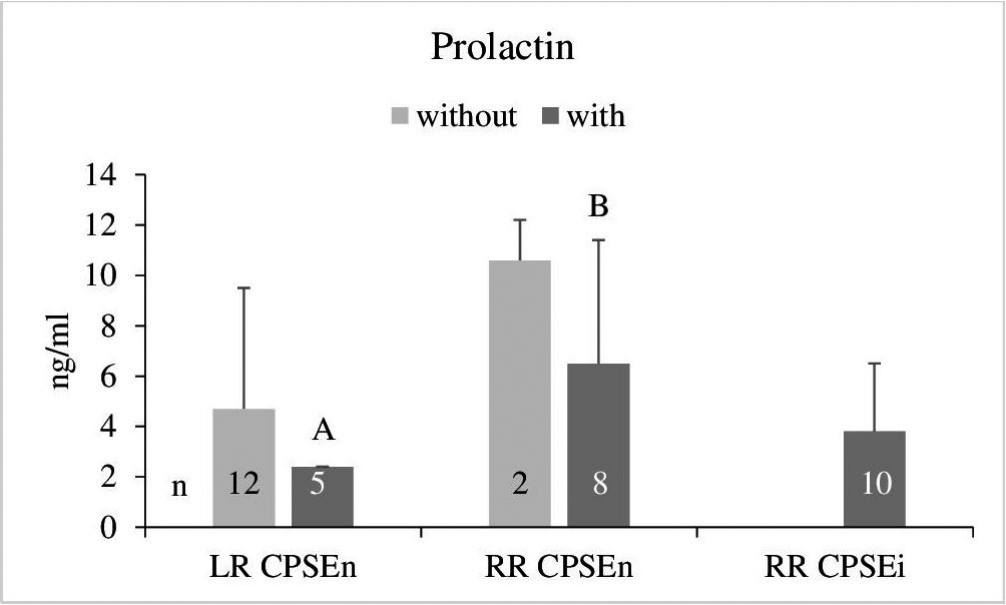
Serum prolactin concentrations (mean ± *SD*) in dogs with normal and increased concentrations of canine prostate‐specific arginine esterase (CPSEn, CPSEi) without and with clinical signs of BPH. A/B: *p* < 0.05 (Wilcoxon test). LR, Labrador Retriever; RR, Rhodesian Ridgeback

Prolactin (PRL) concentrations were 4.0 ± 4.1 ng/ml in the LR dogs and 5.5 ± 4.1 ng/ml in dogs of the RR breed regardless of the CPSE status. The highest mean serum PRL concentration was measured in the two CPSEn RR not showing clinical signs of BPH (Figure 2). With regard to the presence of clinical signs of BPH, PRL concentrations were significantly higher in the CPSEn RR than in the LR dogs (*p* < 0.05). In the CPSEi RR, the mean PRL level was similar to that of the LR dogs (Figure 2).

For the entire group of dogs, a significant correlation of PRL was found with the breed (*r* = 0.39, *p* <.05). A correlation of PRL with CPSE was detected in the RR CPSEi with clinical signs of BPH (*r* = 0.72, *p* < 0.05) (Table 1).

### 3.3 IGF‐1

**FIGURE 3:**
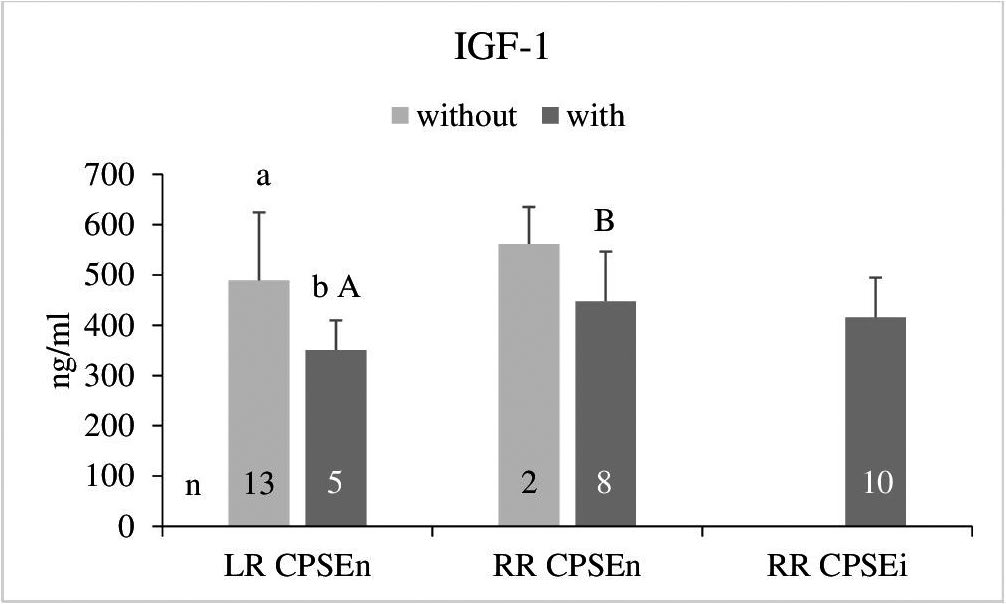
Serum IGF‐1 concentrations (mean ± *SD*) in dogs with normal and increased concentrations of canine prostate‐specific arginine esterase (CPSEn, CPSEi) without and with clinical signs of BPH. a/b: *p* < 0.05 (Wilcoxon test), A/B *p* = 0.06 (Kruskal Wallis test). LR, Labrador Retriever; RR, Rhodesian Ridgeback

Serum concentrations of IGF‐1 were 450.7 ± 133.5 ng/ml in the LR dogs and 443.1 ± 93.4 ng/ml in the entire group of RR. In the dogs showing clinical signs of BPH, IGF‐1 concentrations were generally lower regardless of CPSE status and breed. The difference within the LR group was significant (*p* < 0.05) (Figure 3). Mean IGF‐1 concentrations were slightly higher in the CPSEn RR dogs with clinical signs of BPH than in the corresponding group of LR dogs (*p* = 0.06) (Figure 3).

Negative correlations were found between the IGF‐1 concentration and PGV considering all dogs (*r* = −0.33, *p* < 0.05), the RR CPSEn with clinical signs of BPH (*r* = −0.89, *p* < 0.01) and the entire group of RR with clinical signs of BPH (*r* = −0.47, *p* = 0.05) (Table 1). Except in the latter group, IGF‐1 concentrations were negatively correlated with age (all dogs: *r* = −0.37, *p* < 0.05; RR CPSEn with clinical signs of BPH: *r* = 0.79, *p* < 0.05) (Table 1). For all 38 dogs, a negative correlation was detected between IGF‐1 and CPSE (*r* = −0.36, *p* < 0.05).

### 3.4 Calcidiol/25‐hydroxy vitamin D_3_ (25‐OHD_3_)

**FIGURE 4:**
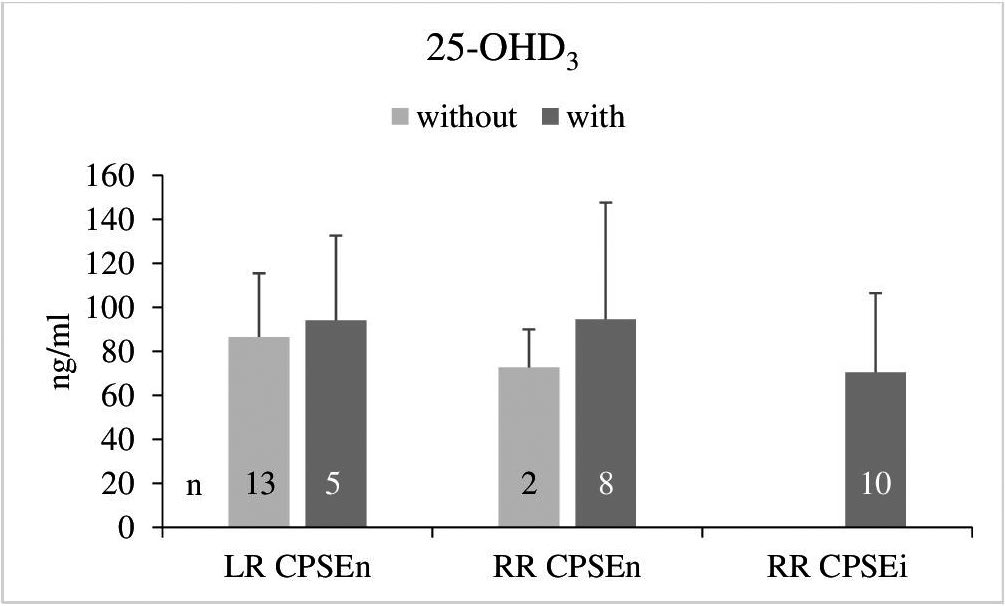
Serum 25‐OHD_3_ concentrations (mean ± *SD*) in dogs with normal and increased concentrations of canine prostate‐specific arginine esterase (CPSEn, CPSEi) without and with clinical signs of BPH. LR, Labrador Retriever; RR, Rhodesian Ridgeback

The overall 25‐OHD_3_ concentrations in blood serum of the LR and RR dogs were 88.7 ± 30.8 and 80.4 ± 42.5 ng/ml, respectively. The 25‐OHD_3_ pattern regarding the CPSE status and the absence or presence of clinical signs of BPH showed no differences between groups except a tendentially (*p* = 0.18) lower mean level in the RR with CPSEi (Figure 4).

Serum 25‐OHD_3_ was negatively correlated with age in the entire group of dogs (*r* = −0.33, *p* < 0.05), in the RR with CPSEi (*r* = −0.64, *p* < 0.05) and in the RR (CPSEn & CPSEi) with clinical signs of BPH (*r* = −0.47, *p* = 0.05) (Table 1).

Analysis of 25‐OHD_3_ in prostatic secretion revealed detectable values only in dogs of the RR breed showing clinical signs of BPH (CPSEn *n* = 4:63.3 ± 50.2 ng/ml, CPSEi *n* = 7:44.4 ± 31.4 ng/ml). In the prostatic secretion of the LR dogs, 25‐OHD_3_ could not be verified. Out of the four affected CPSEn RR dogs, one belonged to age group 18–24 months and one to age group 25–48 months and two belonged to age group 49–72 months. These four dogs showed clinical signs of BPH (HS *n* = 2, SF & HS *n* = 2). In the seven CPSEi RR dogs, the distribution of clinical signs in the age groups was 18–24 months—HS *n* = 1, SF & HS *n* = 1; 25–48 months—HS *n* = 1; 49–72 months—SF & HS *n* = 4.

Due to the fact, that 25‐OHD_3_ was only detectable in the prostatic secretion of RR dogs, a positive correlation existed with breed considering the entire group of dogs (*r* = 0.59, *p* < 0.0001) (Table 1). For the RR dogs with clinical signs of BPH (CPSEn & CPSEi) a close to significant correlation was detected with PGV (*r* = 0.46, *p* = 0.06) (Table 1).

## 4 DISCUSSION

### 4.1 Suitability of the selected dogs with regard to the aims of the study

One important feature of selection was the almost identical body weight with regard to the comparability of the size of the reproductive organs, especially the prostate gland, and the well‐known positive correlation between body weight and plasma IGF‐1 concentration (Eigenmann et al., 1988; Greer et al., 2011; Jaillardon et al., 2011).

Verified euthyroidism was a further essential prerequisite due to the interaction of thyrotropin releasing hormone (TRH) and PRL secretion, demonstrated in both, intact and castrated male beagle dogs (Günzel‐Apel et al., 2009; Koch et al., 2006) as well as in dogs of different breeds (Urhausen et al., 2009). Primary hypothyroidism has also been described as a possible reason for significantly increased IGF‐1 concentrations (Jaillardon et al., 2011).

Regarding the prostate gland status, no one of the dogs showed clinical signs of BPH like sanguineous fluid dripping from the tip of the penis, haematuria, constipation, and difficult urination (Johnston et al., 2001; Krawiec & Heflin, 1992). Association of BPH with chronic prostatitis as frequently found in human (Bostanci et al., 2013) may exist in dogs with a more progressed BPH than was the case in the dogs included in the study (Nizanski et al., 2014). Furthermore, lower urinary tract symptoms like dysuria described to be related to BPH and chronic prostatitis in men (Nickel et al., 2008) is very rare in dogs. It may be observed in case of severe prostatic findings like large cavities and neoplasia (Lopate, 2010). Presumptive diagnosis of chronic prostatitis in dogs is based on low fertility due to poor semen quality (decreased percentage of motile or morphologically normal sperm) (Johnston et al., 2001). Furthermore, chronic prostatitis in dogs is mostly due to bacterial infection (Barsanti & Finco, 1979). Thus, final diagnosis is made by detecting inflammatory exudate and bacterial pathogens in semen and/or prostatic fluid (Barsanti & Finco, 1984; Johnston et al., 2000; Ling et al., 1990). By performing a complete breeding soundness examination, these indications of chronic prostatitis could be ruled out.

In dogs, haemospermia without changes in semen quality is known to be the most common clinical symptom in early stages of BPH and is to be expected alone or combined with sonographic signs in dogs of maximum 6 years of age. The higher incidence of these criteria and of increased CPSE concentrations (Figure 1) as well as the significantly larger PGV in the RR dogs showing clinical signs of BPH compared with the conditions in the LR (RR CPSEn: *p* = 0.06, RR CPSEi: *p* < 0.05) indicate the breed specific predisposition to develop BPH ( ). This is underlined by the correlation of PGV with age both in the RR CPSEn and the entire group of RR with clinical signs of BPH (both *p* <.05) (Table 1). The correlation of PGV with age detected in the LR without clinical signs of BPH (*p* <.001) (Table 1) represents the species‐specific continuous development of clinically asymptomatic BPH.

### 4.2 Prolactin

The mean PRL concentrations measured in the present study result from a single PRL value per dog. Regarding diurnal PRL secretion, Koch et al., (2006) identified in male beagle dogs just a smoothly oscillating baseline without significant pulses over a 6‐hr period, whereas Corrada et al., (2003) detected occasional distinct elevations in individual dogs as well as a circannual rhythmicity of PRL secretion. However, neither study considered the prostate gland status of the dogs.

By means of the PRL serum concentrations, the hypothesized stimulating influence of PRL on the development of BPH could not be clearly shown either in the dogs with clinical signs of BPH or in the predisposed RR breed. A hint towards breed relation is given by the correlation between PRL and breed (*p* < 0.05) (Table 1). The positive correlation between PRL and CPSE detected for the RR CPSEi (*p* < 0.05) (Table 1) may indirectly indicate interaction of PRL and androgens. Prolactin is supposed to raise the uptake of testosterone into the prostate and the intraprostatic testosterone metabolism (Farnsworth et al., 1981). This is supported by experimental studies in rodents. In rats, for example, prolactin has been suggested to work synergistically with testosterone to increase 5α‐reductase activity in both, in vitro and in vivo studies, and induces growth, differentiation and hyperplastic changes of the prostate (Lane et al., 1997; Nevalainen et al., 1996; Reiter et al., 1999; Tangbanluelkal & Robinette, 1993). However, in the study of Kukk (2011), no correlation was found between PRL on the one hand and BPH or linked parameters on the other hand.

As shown in experimental models, PRL acts as a direct growth and differentiation factor for the prostate independent from androgens (Nevalainen et al., 1997; Reiter et al., 1999; Syms et al., 2006). Expression of PRL and its receptor has been verified in epithelial cells of the rat and human prostate gland (Hair et al., 2002; Nevalainen et al., 1997; Ouhtit et al., 1993). As prolactin and its binding sites have also been identified in various tissues including focal glandular hyperplastic cells of the canine prostate (El Etreby & Mahrous, 1979), stimulatory effects of PRL on prostate growth and development of canine BPH can be expected.

### 4.3 IGF‐1

Regarding IGF‐1, the absolute serum concentrations measured in the present study in male LR and RR dogs were on a similar level as analysed in a serum pool of healthy intact beagle dogs and in a group of healthy dogs of different breeds of both genders, respectively (McQuown et al., 2018; Tvarijonaviciute et al., 2011).

A stimulating influence of IGF‐1 on the development of canine BPH as expected with our null hypothesis could not be verified in the present study. However, similar to our results, lower serum IGF‐1 levels were found in human patients with BPH compared with healthy controls (Safarinejad et al., 2011). This agrees with the lower mean IGF‐1 concentrations found in both, the LR (*p* < 0.05) and RR with clinical signs of BPH irrespective of the CPSE status (*p* > 0.05) (Figure 3). It is underlined by the negative correlation between IGF‐1 and PGV detected for the entire group of dogs (*p* < 0.05) and the RR with clinical signs of BPH (CPSEn *p* < 0.01; CPSEn&CPSEi *p* = 0.05) (Table 1). A decline in IGF‐1 over time has been described for both intact male and female dogs regarding a lifespan of several months to >16 years (Greer et al., 2011). The negative correlation of IGF‐1 with age detected in our study, may primarily reflect independence of BPH, as it was verified for the entire group of dogs (*p* < 0.05) and the RR CPSEn with clinical signs of BPH (*p* <.05) but not for the RR CPSEi (Table 1). Furthermore, the negative correlations between IGF‐1 on the one hand and prostate gland volume (all dogs: *p* < 0.05, RR CPSEn with clinical signs *p* < 0.01, RR CPSEn&CPSEi with clinical signs *p* = 0.05) and the serum CPSE concentration (*p* < 0.05) on the other hand (Table 1) contradict a stimulating effect of IGF‐1 on BPH development.

The bioactivity of IGF‐1 within tissues may not only depend on circulating IGF‐1 levels, but also on the local production of IGF‐1 and the presence of IGF‐binding proteins (IGFBPs) (Stattin et al., 2001). Human prostate cells have been demonstrated to express IGF, IGFBPs and the type 1 IGF receptor (Chokkalingam et al., 2002). Among IGFBPs, IGFBP‐3 is the most abundant form, with the highest affinity for IGF‐1 in the circulatory system, binding 75%–90% of circulating IGF‐1 (Lee et al., 2014). IGFBP‐3 is a strong anti‐proliferative protein that provokes apoptosis and inhibits cell proliferation in human prostate cancer. As described for IGF‐1, IGFBP‐3 levels were significantly lower in men with BPH compared with controls (Safarinejad et al., 2011). Moreover, it is possible that circulating androgens may inﬂuence IGF bioactivity levels, particularly because the androgen pathway inﬂuences IGF‐mediated cellular regulation. Androgens have been shown to promote the expression of the type 1 IGF receptor (Vendola et al., 1999).

### 4.4 Calcidiol/25‐hydroxy vitamin D_3_ (25‐OHD_3_)

Serum concentrations of 25‐OHD_3_ in the LR and RR CPSEn without and with clinical signs of BPH did not reveal a relation with BPH. The only indication of a causal connection with BPH, as expected in our null hypothesis, may be assumed in the tendentially (*p* = 0.18) lower 25‐OHD_3_ concentrations in the RR CPSEi (Figure 4). Significant differences, as found in a human study between men with healthy prostate gland and BPH (Elshazly et al., 2017) are missing. Regarding a possible clinical relevance of the findings obtained in the RR CPSEi dogs, the largest prostate gland volume and the presence of clinical signs of BPH must be considered in addition to the increased CPSE. With this in mind, the tendentially lower 25‐OHD_3_ serum concentrations may indicate insufficient supply of vitamin D or breed related changes in vitamin D metabolism. The latter is underlined by the negative correlation between 25‐OHD_3_ and age (*p* < 0.05) as well as by the breed specific verification of 25‐OHD_3_ in the prostatic secretion of the RR (*p* < 0.0001).

It is generally accepted that dogs primarily rely upon dietary intake of vitamin D (How et al., 1994). With regard to health, benign disease and cancer in dogs, relative risk was found to increase with decreasing serum 25‐OHD_3_ concentrations, with a lower limit of 40 ng/ml. Vitamin D sufficiency was defined as serum concentrations of 100–120 ng/ml (Selting et al., 2014). In 292 dogs fed commercial foods from 40 different manufacturers, Sharp et al., (2015) found an overall median serum 25‐OHD_3_ concentration of 67.9 ng/ml ranging from 47.4 to 100.1 ng/ml with significant differences among the manufacturers. In the present study, a similar range of values existed in the majority of dogs. Values above 100 ng/ml were measured in each of six LR and RR dogs (LR CPSEn: without *n* = 3, with *n* = 3; RR CPSEn: with *n* = 3, CPSEi *n* = 3) (Table 2). Lowest values of 15.6–17.0 ng/ml existed only in three 49‐ to 72‐month‐old dogs of the RR breed with clinical signs of BPH (RR CPSEn *n* = 1, RR CPSEi *n* = 2). In humans, vitamin D has gained increasing interest with regard to its anti‐proliferative effects and its role as a mediator of cell differentiation and apoptosis in multiple tissues including the prostate gland (Haussler et al., 1998; Nagpal et al., 2005). Moreover, the vitamin D receptor (VDR) has been isolated and identified in rat and human prostatic tissue (Blomberg Jensen et al., 2011; Holick, 2008; Johnson et al., 1996). Expression of VDR has also been detected in cultured stromal cells derived from the prostate of human BPH patients (Crescioli et al., 2003, 2005; Peehl et al., 1994). The use of vitamin D analogues specific to the VDR in human prostatic tissue has resulted in a significant shrinkage of the enlarged gland in men with benign prostatic hyperplasia (Adorini et al., 2010; Colli et al., 2006; Crescioli et al., 2004). In addition to VDR, normal, cancerous and hyperplastic human prostatic tissue has been shown to express D‐1α‐hydroxylase activity and contain 1,25‐(OH)_2_D_3_, indicating that it is able to synthesize its own intracellularly active 1,25‐(OH)_2_D_3_ from 25‐OHD_3_ (Holick, 2008; Schwartz et al., 1998). Vitamin D receptor was also verified in the canine prostate gland both in vitro and in vivo (Adorini et al., 2007; Kunakornsawat et al., 2004; Taniguchi et al., 2010). Furthermore, cell growth was inhibited by 1,25‐(OH)_2_D_3_ (Kunakornsawat et al., 2004), and the anti‐proliferative effect of VDR agonists was proven in prostate glands and prostatic tissue of Beagles with spontaneous BPH (Adorini et al., 2007; Taniguchi et al., 2010).

**TABLE 2:** Serum 25‐OHD_3_ concentrations (ng/ml) in Labrador Retrievers (LR) and Rhodesian Ridgebacks (RR) with regard to the prostate gland status (CPSEn = normal concentration, CPSEi = increased concentration of CPSE, without and with clinical signs of BPH)

	mean	±*SD*	min‐max	>100 *n*	<20 *n*
LR CPSEn					
without (*n* = 13)	86.6	28.9	48.7–151.4	4	0
with (*n* = 5)	94.1	38.5	48.6–153.6	2	0
RR CPSEn					
without (*n* = 2)	72.7	17.3	60.6–85.0	0	0
with (*n* = 8)	94.6	53.0	15.6–189.3	3	1
RR CPSEi	70.5	36.0	15.7–114.4	3	2

The negative correlation of serum 25‐OHD_3_ with age seen in the entire group of dogs (*p* < 0.05) and especially in the RR dogs showing clinical signs of BPH (RR CPSEi *p* < 0.05, RR CPSEn & CPSEi *p* = 0.05) (Table 1), may indicate an age‐related reduction of the endogenous transformation of vitamin D to 25‐OHD_3_ (Ebeling et al., 1992). This may result in a decrease in the inhibiting influence of 25‐OHD_3_ or 1,25‐(OH)_2_D_3_ on the development of BPH, caused by an age‐related reduction in intraprostatic synthesis of 1,25‐(OH)_2_D_3_ or of prostatic VDR expression leading to an increased resistance of prostatic tissue to 1,25‐(OH)_2_D_3_‐mediated effects. This hypothesis is supported by the missing negative correlation between 25‐OHD_3_ and PGV, as found in healthy men with BPH (Park et al., 2018).

Regarding the detection of measurable 25‐OHD_3_ concentrations in the prostatic secretion of 11 RR dogs (CPSEn *n* = 4, CPSEi *n* = 7), a BPH‐related specificity in the RR breed may be assumed. The close to significant correlation between prostate gland volume and the 25‐OHD_3_ concentrations in the prostatic secretion found in the entire group of RR with clinical signs of BPH (*p* = 0.06) (Table 1) may indicate a reduced transformation to 1,25‐(OH)_2_D_3_ in the enlarged prostate gland and a concomitant increased excretion of 25‐OHD_3_ into the prostatic secretion.

## 5 CONCLUSIONS

The results of the present study show that measuring serum concentrations of PRL and IGF‐1 has no clinical relevance for diagnosis or treatment of canine BPH. From the scientific point of view, studies on the expression of prostatic receptors of PRL and IGF‐1 in dogs are scarce so far, and breed‐related conditions are completely missing. Thus, our results are to be taken as an initiation for further investigations that will delve deeper into the canine prostatic tissue. The apparent causal relationship between the serum 25‐OHD_3_ concentrations and age‐dependent changes in vitamin D metabolism together with the proof of 25‐OHD_3_ in the prostatic secretion of the dogs of the RR breed may indicate (1) the clinical relevance of vitamin D supply for the prevention of BPH, (2) the possibility of treating canine BPH by administering vitamin D or substances involved in the intraprostatic vitamin D metabolism.

To the best of our knowledge, peripheral concentrations of IGF‐1 and 25‐OHD_3_ have not yet been considered regarding their possible clinical relevance with respect to canine BPH.

## AUTHOR CONTRIBUTION

**Franziska Werhahn Beining:** Conceptualization; Data curation; Investigation; Writing‐original draft. **Marion Schmicke:** Investigation; Validation. **Mirja Wilkens:** Investigation; Validation. **Karola Wolf:** Data curation; Investigation. **Karl Rohn:** Data curation; Investigation; Validation. **Anne‐Rose Günzel‐Apel:** conceptualization, supervision, writing‐review & editing.

## ETHICAL STATEMENT

The study was approved by the Lower Saxony State Office for Consumer Protection and Food Safety, Germany (Reference no. 33.19–42502–04‐15/1885). Informed owner consent was obtained.

### PEER REVIEW

The peer review history for this article is available at https://publons.com/publon/10.1002/vms3.514.
